# RAD51 as an immunohistochemistry-based marker of poly(ADP-ribose) polymerase inhibitor resistance in ovarian cancer

**DOI:** 10.3389/fonc.2024.1351778

**Published:** 2024-04-25

**Authors:** Yoo-Na Kim, Kyeongmin Kim, Je-Gun Joung, Sang Wun Kim, Sunghoon Kim, Jung-Yun Lee, Eunhyang Park

**Affiliations:** ^1^ Department of Obstetrics and Gynecology, Institute of Women’s Life Medical Science, Yonsei University College of Medicine, Seoul, Republic of Korea; ^2^ Graduate School of Medicine, Yonsei University College of Medicine, Seoul, Republic of Korea; ^3^ Department of Pathology, Soonchunhyang University, Seoul, Republic of Korea; ^4^ Department of Biomedical Science, College of Life Science, CHA University, Seongnam, Republic of Korea; ^5^ Department of Pathology, Yonsei University College of Medicine, Seoul, Republic of Korea

**Keywords:** ovarian cancer, immunohistochemistry, RAD51, PARP inhibitor, resistance

## Abstract

**Objective:**

Effective functional biomarkers that can be readily used in clinical practice to predict poly(ADP-ribose) polymerase inhibitor (PARPi) sensitivity are lacking. With the widespread adoption of PARPi maintenance therapy in ovarian cancer, particularly in patients with *BRCA* mutation or HR deficiencies, accurately identifying *de novo* or acquired resistance to PARPi has become critical in clinical practice. We investigated RAD51 immunohistochemistry (IHC) as a functional biomarker for predicting PARPi sensitivity in ovarian cancer.

**Methods:**

Ovarian cancer patients who had received PARPi and had archival tissue samples prior to PARPi exposure (“pre-PARPi”) and/or after progression on PARPi (“post-PARPi”) were selected. RAD51 IHC expression was semi-quantitatively evaluated using the H-score in geminin (a G2/S phase marker)- and γH2AX (a DNA damage marker)-positive tissues. A RAD51 H-score of 20 was used as the cutoff value.

**Results:**

In total, 72 samples from 56 patients were analyzed. The median RAD51 H-score was 20 (range: 0–90) overall, 10 (0–190) in pre-PARPi samples (n = 34), and 25 (1–170) in post-PARPi samples (n = 19). Among patients with *BRCA* mutations, RAD51-low patients had better progression-free survival (PFS) after PARPi treatment than RAD51-high patients (*P* = 0.029). No difference was found in PFS with respect to the genomic scar score (*P* = 0.930). Analysis of matched pre- and post-PARPi samples collected from 15 patients indicated an increase in the RAD51 H-score upon progression on PARPi, particularly among pre-PARPi low-RAD51-expressing patients.

**Conclusion:**

RAD51 is a potential functional IHC biomarker of *de novo* and acquired PARPi resistance in *BRCA*-mutated ovarian cancer and can be used to fine-tune ovarian cancer treatment.

## 1 Introduction

Poly(ADP-ribose) polymerase inhibitor (PARPi), which is a targeted agent based on synthetic lethality, exploits preexisting defects in the homologous recombination (HR) pathway (1). PARPi therapy has significantly enhanced progression-free survival (PFS) in ovarian cancer patients, as evidenced by first-line maintenance studies. The SOLO1 trial demonstrated benefits in patients with *BRCA* mutations, while the PAOLA1 trial extended these findings to those with wild-type *BRCA* (2). However, there remain two unmet clinical needs. The first need is the identification of patients with *de novo* resistance, as approximately half of the patients with ovarian cancer who receive PARPi treatment in a front-line setting experience disease progression within five years, despite *BRCA* mutations (3). The second need is the identification of acquired resistance mechanisms, including HR repair (HRR)-dependent and -independent mechanisms, in patients who have progressed on PARPi therapy (4–7), as these mechanisms have been studied mostly in preclinical models. Because patients who progress on PARPi do not respond well to subsequent treatments, studies on PARPi resistance are of utmost importance (8).

In clinical settings, the response to PARPi therapy varies greatly, even among patients with *BRCA* mutations, who are expected to respond well. Therefore, genomic markers other than *BRCA* mutations, namely HR deficiency (HRD) has been studied. Of various methods to detect HRD, approaches with genomic sequencing method include various copy number alteration-based methods such as loss of heterozygosity (LOH), telomeric allelic imbalances (TAI), and large-scale transitions (LST) (9) and single nucleotide variant-based measures such as signature 3 (10). However, a disadvantage of these genomic biomarkers is that they remain static throughout PARPi treatment (11). For instance, genomic LOH and the HRD score remained unchanged after progression on PARPi (12, 13). The biological status of longitudinal tumor samples is likely to change dynamically. For example, HRR (i.e., the development of an HR-proficient phenotype) is a resistance mechanism that can be induced by platinum-based chemotherapy or PARPi exposure (14). Such restoration can occur at any time during PARPi treatment and as early as immediately after first-line chemotherapy (15–17). Therefore, identifying a functional marker that reflects HR status at any time point during treatment is crucial.

RAD51 is a key effector in the HR-mediated DNA repair pathway (18). RAD51 is recruited onto the DNA break by BRCA2 to form homopolymeric filaments that invade homologous chromatids and use them as templates for HRR. HR proficiency can be assessed using RAD51 immunofluorescence (IF) staining, which detects RAD51 filaments as distinct foci in the nucleus. First described in primary cultures of ovarian tumor cells (19), RAD51 IF staining has been primarily studied in breast cancer (20, 21). In earlier studies, live cells were subjected to external DNA damage, whereas in later studies biopsy specimen (22) and formalin-fixed paraffin-embedded (FFPE) samples were also utilized (23). As a practical alternative to IF staining, RAD51 foci assessment using immunohistochemistry (IHC) was also suggested (15, 24, 25). Further enhancing clinical applicability, the requirement for external DNA damage was eliminated, as recent studies proposed that endogenous DNA damage was shown to be sufficient for RAD51 assays (23). However, RAD51 IHC using patient-derived tissue samples in PARPi context (i.e., before PARPi and its time-lagged changes after progression on PARPi) has not been reported to date. This study aimed to investigate the potential of RAD51 IHC in predicting *de novo* and acquired resistance in patients with ovarian cancer who had received PARPi.

## 2 Methods

### 2.1 Patient recruitment

Among patients with ovarian cancer who had received PARPi between September 2017 and June 2022 at the Yonsei Cancer Center (Seoul, Korea), those with archival FFPE tissue samples prior to PARPi exposure (“pre-PARPi”) and/or after progression on PARPi (“post-PARPi”) were selected. The study was approved by the Institutional Review Board of Yonsei University (approval number 4-2020-0386) and adhered to the principles of the Declaration of Helsinki. Informed consent was obtained from all participants.

### 2.2 Histological and IHC analysis

For histologic analysis, hematoxylin and eosin-stained tumor-containing slides were independently reviewed by gynecologic pathologists and a representative FFPE tumor tissue block was selected.

For IHC analysis, we used three markers: RAD51 for defining the HR status, geminin for identifying cells in the G2/S cell-cycle phase, and γH2AX as a DNA double-strand break marker (26). The following antibodies were used for the staining: RAD51 (1:1,000, clone 14B4; GeneTex, Irvine, CA, USA), geminin (1:1,000, clone 10802-1-AP; ProteinTech, Chicago, IL, USA), and γH2AX (1:10,000, clone JBW301; Sigma-Aldrich, Dorset, UK). Approximately 4-μm-thick sections of FFPE blocks were immunostained using a Ventana BenchMark XT automated stainer (Ventana Medical Systems, Tucson, AZ, USA) and using the UltraView Universal DAB Detection Kit (Ventana Medical Systems). Positive and negative controls were stained concurrently to validate the staining method. Germ cells from normal testicular testis tissue was used as a positive control for RAD51 and geminin, while normal palatine tonsil tissue was the positive control for γH2AX. Tests were also conducted without the primary antibody to act as negative controls for all antibodies.

### 2.3 Interpretation of HR status-related markers

Cases with low cellularity (<300 tumor cells/high-power field) were excluded. To evaluate cells in the G2/S phase and with DNA damage, tumors with <25% γH2AX-positive cells or <3% geminin-positive cells were excluded (20). For RAD51 IHC analysis, nuclear expression was evaluated using the H-score method, which is a semi-quantitative system with a 0–300 score range. The percentage of positive cells (0–100%) was multiplied by the dominant staining intensity score. Scores were defined as follows: 0, no staining; 1, barely detectable staining; 2, distinct brown staining; and 3, strong dark brown staining. Applying the optimal cutoff for RAD51 expression determined based on maximally selected rank statistics formulated using the Contal and O’Quigley method, RAD51 H-score <20 was defined as RAD51-low and RAD51 H-score ≥20 was defined as RAD51-high (27). This binary definition was used in all analyses, unless otherwise specified. All slides were evaluated by two experienced pathologists (E.P. and K.K.) in a blinded manner. If discrepancies occurred, consensus was reached.

### 2.4 Germline *BRCA1/2* sequencing, tumor sequencing, and circulating tumor DNA analyses

*BRCA1/2* genetic testing was performed using genomic DNA from peripheral blood samples. Sanger sequencing analysis was performed using a 3730 DNA Analyzer with a BigDye Terminator v3.1 Cycle Sequencing Kit (Applied Biosystems, Foster City, CA, USA) and the Sequencher 5.3 software (Gene Codes, Ann Arbor, MI). Next-generation sequencing was performed on a proportion of patients using a custom panel, including *BRCA1* and *BRCA2*, on a MiSeq sequencer (Illumina, San Diego, CA, USA) using the MiSeq Reagent Kit v2 (300 cycles). Bioinformatic analysis was performed using the Burrows–Wheeler Aligner, Genome Analysis Toolkit, Ensembl Variant Effect Predictor, and a custom pipeline. Experienced geneticists made the final interpretations.

Whole-exome data were obtained from fresh frozen or FFPE samples. Genomic scarring was estimated by determining the copy number alterations in the sequencing data using Sequenza-utils (v.3.0.0) (28) based on LOH, TAI, and LST estimated using the R package scarHRD (v.0.1.1) (29). The sum of these values was defined as the genomic scar score (GSS). Details of the sequencing methodology have been described previously (12).

Cell-free DNA extracted from serially collected whole blood samples was used for next-generation sequencing using an Illumina NovaSeq 6000 System. The sequencing library was prepared using the Library Prep Reagent for Illumina (Dxome, Seoul, Korea). Target enrichment was performed using the TMB 500 panel, whereby 531 cancer-related genes were targeted (Dxome) and analyzed using the PiSeq algorithm (Dxome). All variants were classified into a four-tiered system based on the standards and guidelines of the Association of Molecular Pathology/American Society of Clinical Oncology/College of American Pathologists. Details of the ctDNA preprocessing steps have been described previously (30).

Based on the ctDNA mutation profiles from pre- and post-treatment matched samples, mutations specifically present in post-treatment samples (i.e., not present in pre-treatment samples) were identified in each patient (31) and used to identify resistance mechanisms. As performed in our previous approach (30), individual genes in the TMB500 panel were individually reviewed to determine their implication in PARPi resistance. Among patients who were tested for both RAD51 IHC and ctDNA, resistance mechanisms were classified into HR restoration, replication fork stabilization, survival upregulation, target loss, drug efflux, and *BRCA* reversion (30).

### 2.5 Collection of clinical variables

Clinical variables were evaluated to assess RAD51 IHC efficacy. Basic clinical variables, including histology, International Federation of Gynecology and Obstetrics stage, and *BRCA* status, were collected. Clinical variables associated with PARPi, such as drug type, treatment setting, and line of treatment, were collected. The treatment setting was defined as maintenance if PARPi was administered immediately following a complete or partial response to chemotherapy, or as salvage if PARPi was administered as a treatment without any preceding chemotherapy. Medical records were reviewed for PARPi start date, disease recurrence date, or PARPi end date for causes other than recurrence or death. Disease recurrence was determined based on a radiological assessment, which was performed every three months for the first two years and every six months thereafter. PFS was defined as the period from the PARPi start date to the date of disease progression or PARPi end date, whichever occurred first. Patients who did not experience a second disease progression or died were considered censored.

### 2.6 Statistical analysis

A standard box-and-whisker plot was used to summarize the continuous data by indicating the median, the first and third quantiles within the box, whiskers extending to 1.5 times the interquartile range, and any outlier points. Statistical significance was calculated using Fisher’s exact test or the chi-squared test for categorical variables and Student’s *t*-test for continuous variables. The Shapiro-Wilk test was used to check the normalization assumption; based on these results, data are presented as either the median with range or the mean with standard deviation, as appropriate. The Kaplan–Meier method was used for PFS analysis. A cox proportional hazards regression model was used to evaluate the impact of RAD51 H-score as well as other relevant prognostic variables on disease progression on PARPi. Statistical significance was set at *P* < 0.05. All statistical analyses were performed using R v.4.0.3 (R Foundation for Statistical Computing, Vienna, Austria).

## 3 Results

**Figure 1:**
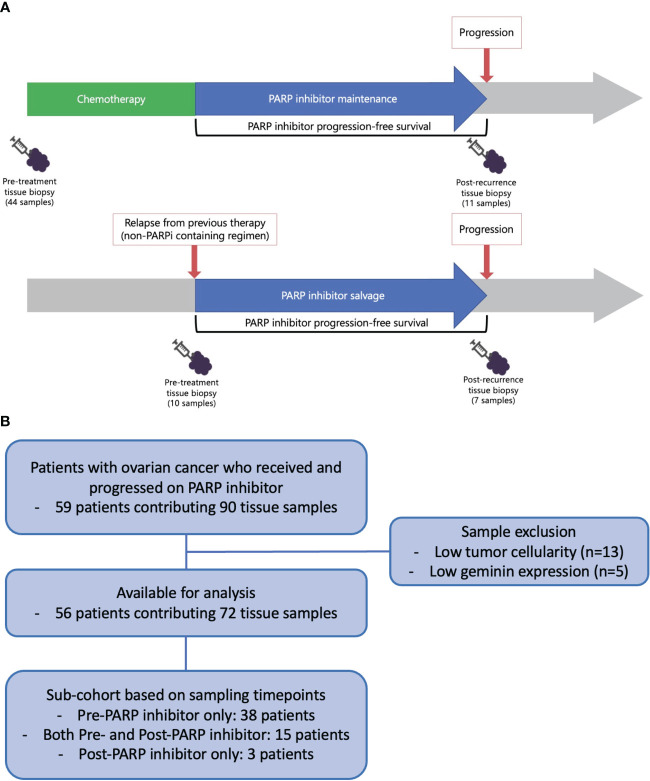
**(A)** Study schema and **(B)** flow chart.

Ninety tissue samples were collected from 59 patients (**Figure 1A**). Archival tissue samples were used whenever available (44/54 pre-treatment tissues were archival), and pre-PARPi tissues were available for most patients who had received PARPi in the maintenance setting. As the HR pathway is only active after DNA double-strand breaks in proliferating cells, we evaluated RAD51 IHC after the exclusion of low-γH2AX (DNA damage marker)- or low-geminin (G2/S phase marker)-expressing tumor samples. After excluding 18 samples with very low tumor cellularity and five samples with low geminin expression, 72 samples from 56 patients were analyzed (**Figure 1B**). Patients’ demographics are summarized in **Table 1**. Most patients had advanced-stage disease, with high-grade serous histology. Most patients had *BRCA* mutations (94.6%) and received olaparib (67.9%) in the maintenance setting (78.6%).

**Table 1:** Patient demographics.

Variable	Patients (n = 56)
Histology	
High-grade serous carcinoma Endometrioid	55 (98.2%) 1 (1.8%)
Stage	
I II III IV	1 (1.8%) 1 (1.8%) 23 (41.1%) 31 (55.3%)
*BRCA* status	
Mutated Wild-type	53 (94.6%) 3 (5.4%)
PARPi type	
Olaparib Niraparib Rucaparib	38 (67.9%) 13 (23.2%) 5 (8.9%)
PARPi setting	
Maintenance Salvage	44 (78.6%) 12 (21.4%)
PARPi line of treatment	
1 2 3 +	13 (23.2%) 22 (39.3%) 21 (37.5%)

**Figure 2:**
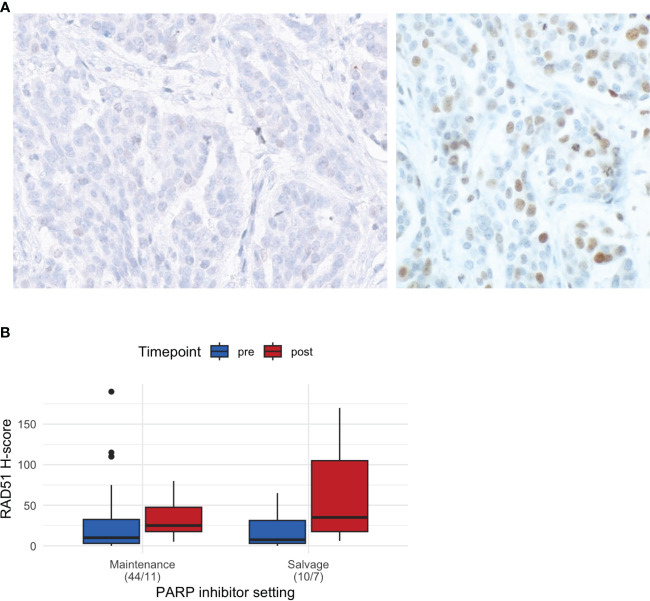
Basic characteristics of RAD51 IHC. **(A)** Representative images of RAD51-low (<20) and RAD51-high (≥20) tumors. **(B)** RAD51 H-score in all samples, showing the differences in the H-score (median and first and third interquartile range in the boxplot) in pre- and post-PARPi samples, with stratification by treatment setting. IHC immunohistochemistry.

**Figure 3:**
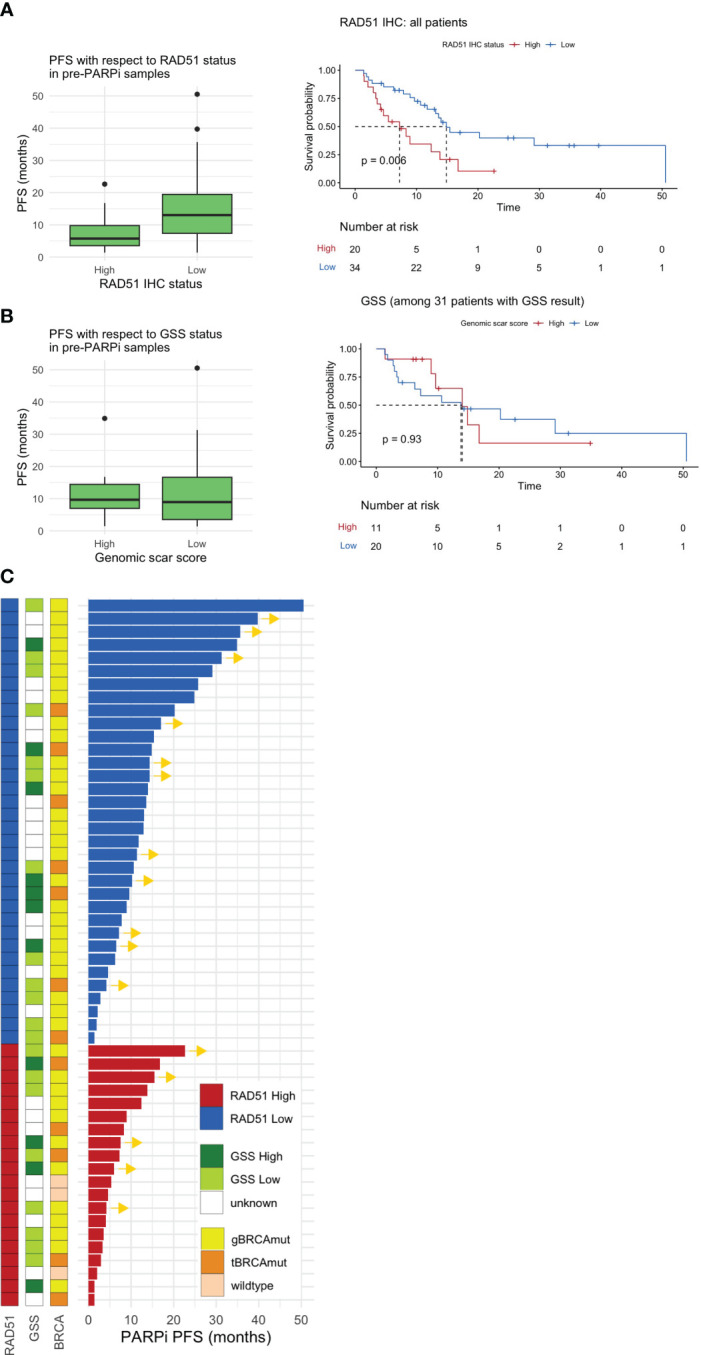
Pre-PARPi analysis. PFS with respect to the **(A)** RAD51 H-score and **(B)** GSS based on pre-PARPi samples. A standard box-and-whisker plot representation, showing the median and first and third interquartile range shown inside the boxplot and outliers that span beyond 1.5 times the interquartile range (i.e., the whiskers) are shown as dots. Kaplan-Meier curve shows PFS with respect to each stratification in all comers. Of note, all patients with GSS score were *BRCA* mutated. **(C)** Swimmer plot showing PARPi PFS stratified by RAD51 IHC status, the GSS, and *BRCA* status. PFS progression-free survival; GSS genomic scar score; IHC immunohistochemistry.

Representative images of RAD51, geminin, and γH2AX IHC are shown in **Figure 2A** and **Supplementary Figure S1.** The median RAD51 H-score was 15 (range: 0–90) overall, 10 (0–190) in the pre-PARPi samples (n = 54), and 27.5 (5–170) in the post-PARPi samples (n = 18). Stratified by treatment setting, a trend of higher RAD H-score in post-PARPi sample was observed (**Figure 2B**). In maintenance, the median RAD51 score was 10 (0 – 190) in pre-PARPi samples and 25 (5 – 80) in post-PARPi samples (*P* = 0.071); in salvage, the median RAD51 score was 7.5 (0 – 65) in pre-PARPi samples and 35 (6 – 170) in post-PARPi samples (*P* = 0.063). Next, the pre-PARPi RAD51 IHC results were investigated with respect to the PARPi response (**Figure 3**).

In all patients, the RAD51-low patients had better PFS (median 7.23 months, 95% CI: 4.14 to not reached) than RAD51-high patients (median 14.9 months, 95% CI: 13.11 to not reached) (*P* = 0.006) (**Figure 3A**). Further subgroup analysis highlighted that a similar trend was observed in *BRCA*-mutated patients (*P* = 0.029) and those who received PARPi in the maintenance setting (*P* = 0.004) (**Supplementary Figure S2**). Multivariable cox proportional hazards regression model with RAD51 H-score as well as other prognostic variables such as stage and *BRCA* status demonstrate that RAD51 H-score and *gBRCA* status were independent predictors of PARPi response, when given in the maintenance setting, but not in salvage setting (**Supplementary Figure S3**). In contrast to RAD51 H-score, GSS, which was available for 31 patients, was not predictive of PARPi response (**Figure 3B**).

Further analysis of the GSS with respect to RAD51 status indicated that RAD51-low patients were more likely to exhibit a trend of elevated GSS than RAD51-high patients (**Supplementary Figure S4**). Next, the sub-parameters used to calculate the GSS (i.e., LOH, TAI, and LST) were investigated (**Supplementary Figure S4**). We found a significant negative correlation between LST and the RAD51 H-score (*P* = 0.006) and a trend of negative correlation between LOH and TAI. The overall spectrum of PARPi PFS with respect to the GSS, *BRCA* status, and RAD51 IHC status is shown in **Figure 3C**.

**Figure 4:**
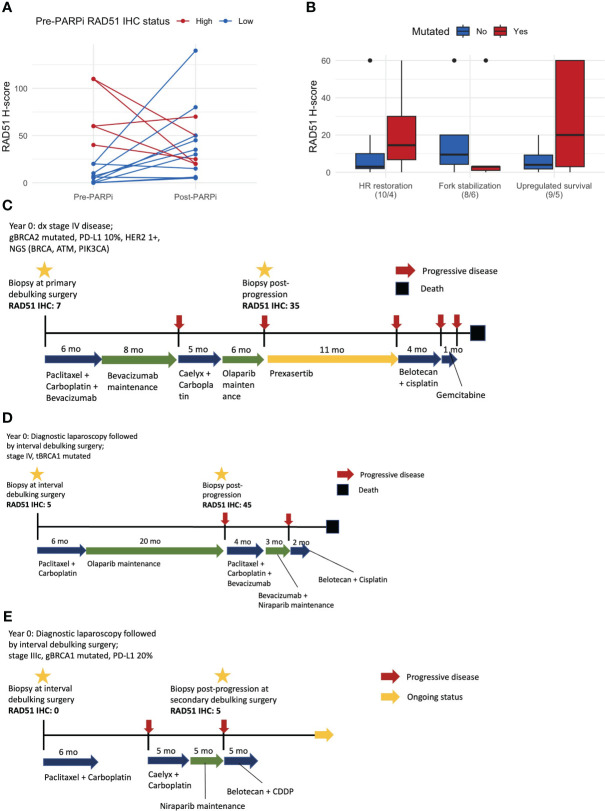
Matched pre- and post-PARPi sample analysis. **(A)** Changes in the RAD51 H-score after progression on PARPi. At each time point, we assessed one representative tissue and evaluated the IHC expression using a whole slide image. Thus, each of the 15 patients with matched samples contributes two points to this line plot. **(B)** RAD51 H-score with respect to the PARPi resistance mechanism determined based on post-PARPi ctDNA samples. **(C)** Patient #1, whose RAD51 IHC status changed from low to high, exhibited an exceptionally good response to a CHK1/CHK2 inhibitor. **(D)** Patient #2, who underwent PARPi treatment for a long period, converted from a RAD51-low to a RAD51-high status and did not respond to subsequent therapy. **(E)** Patient #3 did not demonstrate changes in the RAD51 IHC status; this patient exhibited a partial response to subsequent chemotherapy, which is currently ongoing. ctDNA circulating tumor DNA; IHC immunohistochemistry.

Matched pre- and post-PARPi samples were obtained from 15 patients. The RAD51 H-score increased in 9/15 (60%) patients after exposure to and progression on PARPi. Among 10 patients with RAD51-low pre-treatment, the RAD51 H-score increased after progression on PARPi in eight patients (80%), including a conversion from low to high RAD51 expression in six patients (60%) (**Figure 4A**). Fourteen patients had pre- and post-PARPi ctDNA available. Based on patient-specific assessment of mutations in the pre- and post-PARPi ctDNA, mutations that newly occur in post-recurrence setting were used to classify acquired resistance mechanism. In each patient, the presence or absence of each mechanism (i.e., HR restoration, fork stabilization, and upregulated survival) was ascertained, and the corresponding RAD51 H-scores are shown as boxplots (**Figure 4B**). Based on the matched ctDNA analysis, patients who had HR restoration as a resistance mechanism showed a trend of higher RAD51 H-scores (**Figure 4B**). The mutational spectrum and classification of resistance mechanism are shown in **Supplementary Figure S5**. Out of 14 patients who were tested for both RAD51 IHC and pre- and post-PARPi ctDNA, 6 patients showed alteration in HR restoration-associated genes, including 4 patients who harbored acquired mutation in these genes (i.e., post-PARPi specific alteration).

Examples of these cases are shown in **Figure 4**. Patient #1 had stage IV disease, germline *BRCA1* mutations, and pathogenic *BRCA*, *ATM*, and *PIK3CA* mutations (**Figure 4C**). Matched sample analysis revealed a change in the RAD51 IHC status from low to high (H-score: 7 → 35). This patient showed an exceptionally good response to prexasertib, a CHK1/CHK2 inhibitor, as the third-line therapy. Patient #2 had stage IV disease and tumor *BRCA1* mutations (**Figure 4D**) and was administered PARPi as a frontline maintenance therapy for 20 months until disease progression, when the RAD51 IHC status changed from low to high (H-score: 5 → 45). The response to subsequent chemotherapy or PARPi retreatment was poor. Patient #3 had stage IIIC disease and germline *BRCA1* mutations (**Figure 4E**). Matched sample analysis showed that the RAD51 status did not significantly change (H-score: 0 → 5) after progression on PARPi. This patient exhibited a partial response to third-line platinum-based chemotherapy, and treatment is ongoing.

## 4 Discussion

### 4.1 Summary of the main results

The ability to functionally assess HR status, besides germline *BRCA* mutations or genomic scars, is clinically important in *de novo* resistance, which affects the initial response to PARPi therapy, and in acquired resistance, which has implications for subsequent therapy after disease progression on PARPi. We investigated both aspects in tissue samples using RAD51 IHC, which is highly convenient and cost-effective and can, therefore, be readily incorporated into clinical settings. Our data showed that RAD51 IHC status, unlike the GSS, effectively predicted the response of patients with *BRCA* mutations to PARPi therapy. Furthermore, in matched pre- and post-PARPi samples, the HR capacity indicated by the RAD51 H-score increased post-progression, suggesting the potential for stratification of post-progression therapy in the PARPi era.

### 4.2 Results in the context of published literature

Several studies have functionally assessed HR based on RAD51 expression, mostly in breast cancer (15, 20–24). In ovarian cancer, one of the earliest studies examined RAD51 IF expression after irradiating patient-derived primary cells to predict primary chemotherapy response and survival (32). However, the research in RAD51 foci assessment for ovarian cancer has been rapidly evolving, partly triggered by the need to make the assay more accurate and practical for clinical use. For instance, Compadre et al. showed the feasibility and consistency of RAD51 IF across different sample types, such as cell lines, organoids, and FFPE samples (33). Pikkussari et al. assessed RAD51 IF across different time points in newly diagnosed ovarian cancer patients receiving neoadjuvant chemotherapy (34). Both studies attempted automate the counting process. An alternative to the IF-based approach is IHC, which has also been explored in ovarian cancer. For instance, Hoppe et al. conducted one of the largest studies with quantitative IHC in ovarian cancer, using multispectral imaging and an automated approach to study RAD51 foci in FFPE tissues from a platinum monotherapy trial (35). These studies suggest the need to study RAD51 as biomarker, specifically in the context of PARPi treatment. A recent study by Guffanti et al. showed that RAD51 foci with IF correlates with olaparib response in PDX models (36). However, to the best of our knowledge, no study has examined RAD51 IHC expression and its time-lagged changes in FFPE samples from patients with ovarian cancer treated with PARPi.

Besides assessing RAD51 IHC, we analyzed its correlation with the GSS in patients who underwent both tests. RAD51 IHC status outperformed the GSS in accurately predicting the response to PARPi. The limitations of genomic markers, failure to reflect longitudinal and dynamic changes in HR capacity and need for real-time functional markers have been recognized (37). Exposure to three cycles of neoadjuvant therapy can modulate the HR status (11). The degree of HR capacity in each patient may differ after first-line platinum-based chemotherapy immediately before PARPi therapy initiation. Nevertheless, a comparison between the GSS and RAD51 H-score indicated that a RAD51-low status was associated with a high GSS, both being associated with a favorable response to PARPi. Furthermore, the RAD51 H-score was negatively correlated with individual GSS components, particularly LST. Further functional and biological studies may help elucidate the reason for this observation.

Notably, our data suggested that the RAD51 IHC status can identify poor responders to PARPi among patients with deleterious *BRCA* mutations who are expected to respond well to PARPi monotherapy. Currently approved biomarker testing, including the Myriad HRD test, considers all *BRCA*-mutated patients as HR-deficient; that is, currently, the *BRCA* status overrides any other test aiming to identify favorable candidates for PARPi therapy. However, our findings suggest that identifying poor responders to PARPi may be equally important, especially considering that patients with high RAD51 expression account for approximately one-third of patients with *BRCA* mutations. Patients with high RAD51 expression before treatment may benefit from PARPi-based combination therapies, such as PARPi combined with anti-angiogenic agents (38), immune checkpoint inhibitors (39), or HR inhibitors (40, 41).

Using matched pre- and post-PARPi- samples, we observed an increase in the HR capacity, albeit with varying magnitudes. This trend was particularly pronounced in patients with low pre-treatment values. The overall increasing trend is expected from the implication of acquired HR proficiency as part of the PARPi resistance mechanism (4). In case of a steep increase in the RAD51 H-score upon PARPi progression, as demonstrated in the exemplary cases, agents other than PARPi or platinum-based chemotherapy, such as targeted therapy, may help improve the outcome of subsequent therapy. Conversely, approximately one-third of the cohort exhibited high pre-PARPi RAD51 H-scores. This suggested that HR restoration could have occurred before PARPi administration due to factors such as prior chemotherapy or other potential mechanisms of *BRCA* reversion (16, 17). In these patients with high pre-PARPi RAD51 H-scores, RAD51 H-score did not necessarily increase post-treatment, with several instances showing a decreasing trend. We postulate that in a heavily pre-treated setting or in instances where the pre-PARPi RAD51 H-score is already high, the post-PARPi RAD51 H-score will be less interpretable because of confounders. To better understand the dynamics, we think that further prospective studies with more patients with matched samples, involving serial collection of pre- and post-PARPi samples starting from the initial diagnosis and continuing through various treatment stages, are needed.

### 4.3 Strengths and weaknesses

The limitations of this study include the small sample size and its retrospective nature. Given that RAD51 expression was evaluated only in proliferating cells that had DNA double-strand breaks, 20% of the samples were excluded from analysis. These failed samples indicated inherent biological limitations that can be expected from RAD51 IHC. Therefore, the sample size was smaller than expected, and only a subgroup of patients underwent both RAD51 IHC and GSS tests or had matched samples. Moreover, the GSS was based on WES of FFPE tissue, which is not a clinically established method. We based the cutoff of 42 on our previous experience with this method (12, 42), but this cutoff is still arbitrary. Therefore, any conclusions which relate to GSS can only be considered as hypothesis-generating. In the future, a more rigorous approach to assess HRD status should be taken. For example, a more established methods such as Myriad HRD test or HRDetect, or simultaneous testing of different HRD testing approaches could be implemented to get a robust assessment of GSS/HRD status. We assessed bio-banked tissues of all patients with ovarian cancer who received PARPi at our institution over a specific timeframe. In cases where several biopsy specimens were obtained at a single timepoint, we utilized one representative tissue with the largest tumor component. Given the retrospective nature of the study design, there may have been a selection bias.

### 4.4 Perspectives for future research

Given the expanding therapeutic potential of PARPi beyond ovarian cancer, it will be interesting to validate the RAD51 IHC test in other cancer types, such as breast, prostate, and pancreatic cancer. Furthermore, a dedicated functional study, which simultaneously assesses RAD51 via IHC and conducting RNAseq on newly collected, prospectively obtained samples, would yield more definitive insights. Once validated, the RAD51 IHC can be applied to a prospective trial to help stratify patients identify patients who may not respond well to PARPi despite the presence of *BRCA* mutation and provide clues for the choice of subsequent therapy after progression on PARPi.

## 5 Conclusions

To the best of our knowledge, our study is the first to report on RAD51 IHC as a functional marker of HR restoration for *de novo* and acquired PARPi resistance in ovarian cancer. RAD51 IHC, which is convenient, cost-effective, and can be easily assessed with a light microscope used for routine pathological practices, has the potential to be incorporated in the management of ovarian cancer.

## Data availability statement

The raw data supporting the conclusions of this article will be made available by the authors, without undue reservation.

## Ethics statement

The studies involving humans were approved by IRB of Yonsei University 4-2020-0386. The studies were conducted in accordance with the local legislation and institutional requirements. Written informed consent for participation was not required from the participants or the participants’ legal guardians/next of kin in accordance with the national legislation and institutional requirements.

## Author contributions

Y-NK: Conceptualization, Formal analysis, Investigation, Visualization, Writing – original draft. KK: Conceptualization, Formal analysis, Investigation, Methodology, Visualization, Writing – review & editing. J-GJ: Methodology, Writing – review & editing. SWK: Supervision, Writing – review & editing. SHK: Supervision, Writing – review & editing. J-YL: Conceptualization, Investigation, Supervision, Writing – review & editing. EP: Conceptualization, Investigation, Methodology, Supervision, Writing – review & editing.
