# Supplementary material for: RAD51 as an immunohistochemistry-based marker of poly(ADP-ribose) polymerase inhibitor resistance in ovarian cancer
Source: Front Oncol. 2024 Apr 25;14:1351778. doi: 10.3389/fonc.2024.1351778 (PMC11079140; doi:10.3389/fonc.2024.1351778)

Supplementary Material

**Table of Contents**

**Supplementary Figures**

Fig. S1. Representative immunohistochemical staining for geminin and γH2AX expression.………..1

Fig. S2. Kaplan–Meier curve analysis……………………………….….……………………………..2

Fig. S3. Multivariate analysis………………………………………………………………………….3

Fig. S4. Association between RAD51 immunohistochemistry status and the genomic scar score……4

Fig. S5. Mutation spectrum and classification of resistance mechanism ……………………………..5

**Supplementary Fig. S1.** Representative images of immunohistochemical (IHC) staining for geminin and γH2AX expression. (A) Geminin-negative (<40 positive tumor cells) and -positive (≥40 positive tumor cells). (B) γH2AX-negative (<25% in tumor cells) and -positive (≥25% tumor cells).


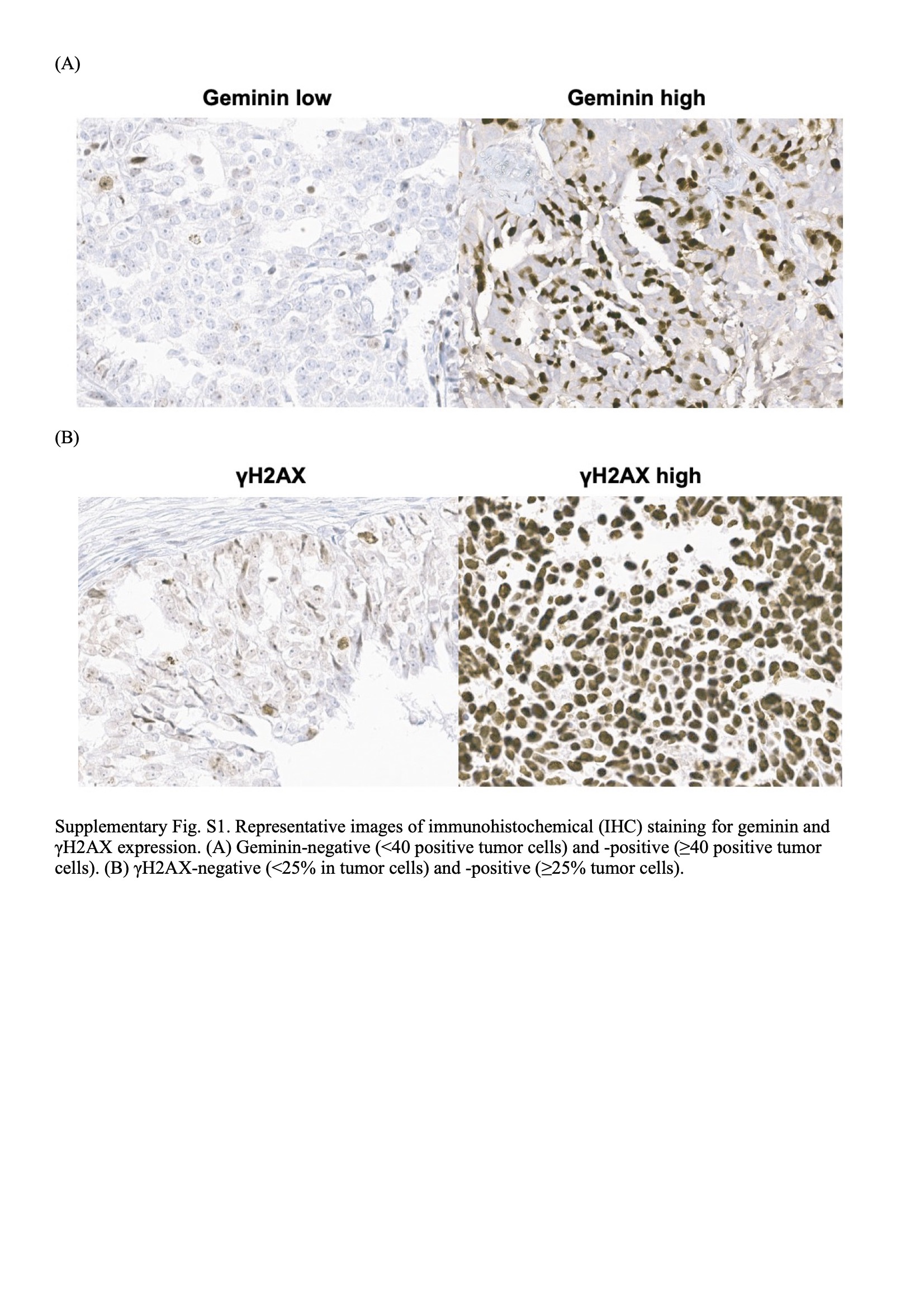


**Supplementary Fig. S2.** Kaplan–Meier curve analysis of progression-free survival (PFS) according to RAD51 IHC status in (A) all patients, (B) patients in the maintenance setting, and patients receiving PARPi (C) in the 1st or 2nd line of treatment or (D) in 3rd or later line of treatment. (E) Kaplan–Meier curve analysis of PFS according to genomic scar score (GSS) in patients in the maintenance setting.


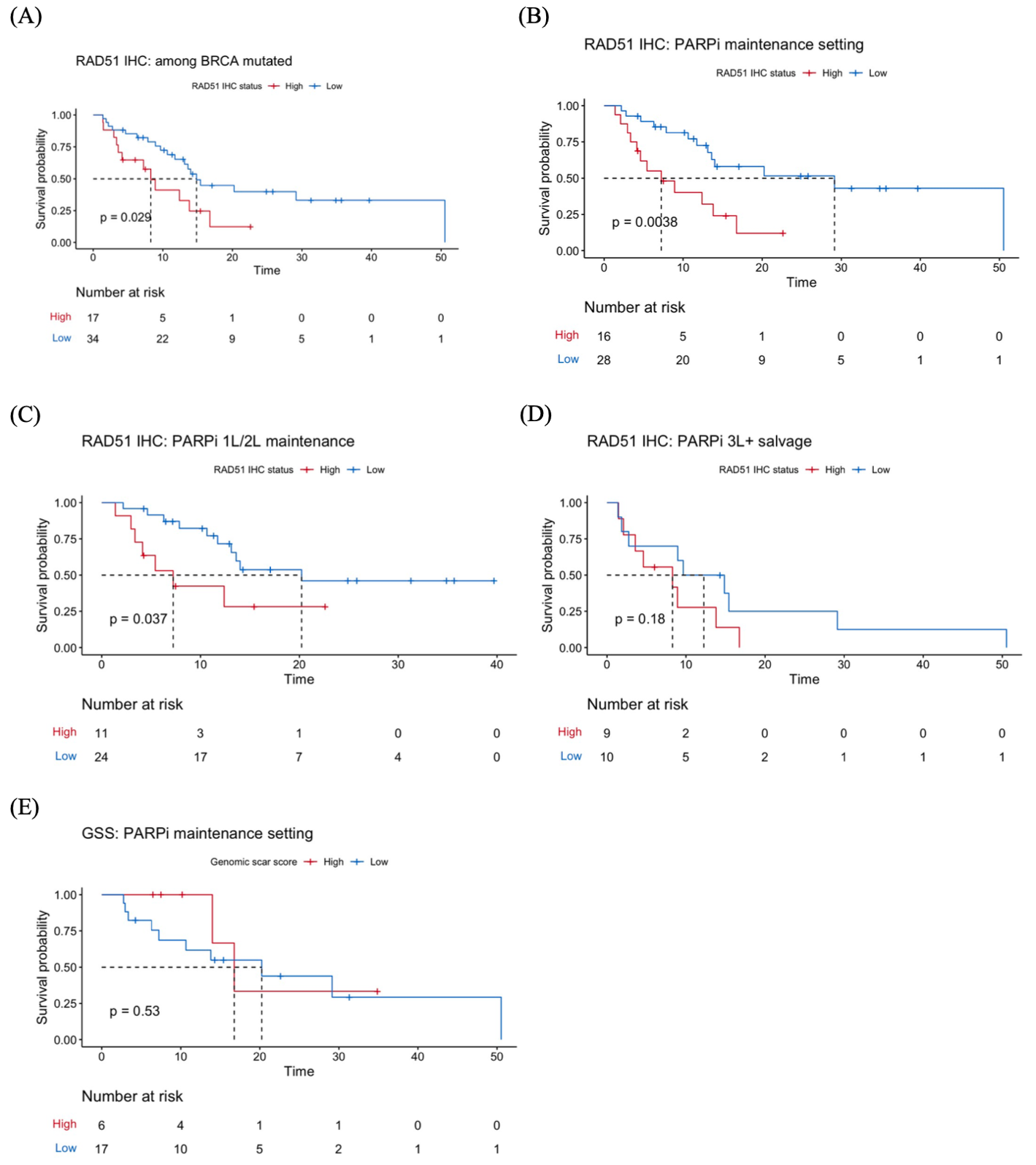


**Supplementary Fig. S3.** Multivariable cox proportional hazards regression analysis in (A) all patients, (B) those receiving PARPi in maintenance setting, and (C) salvage setting.

**
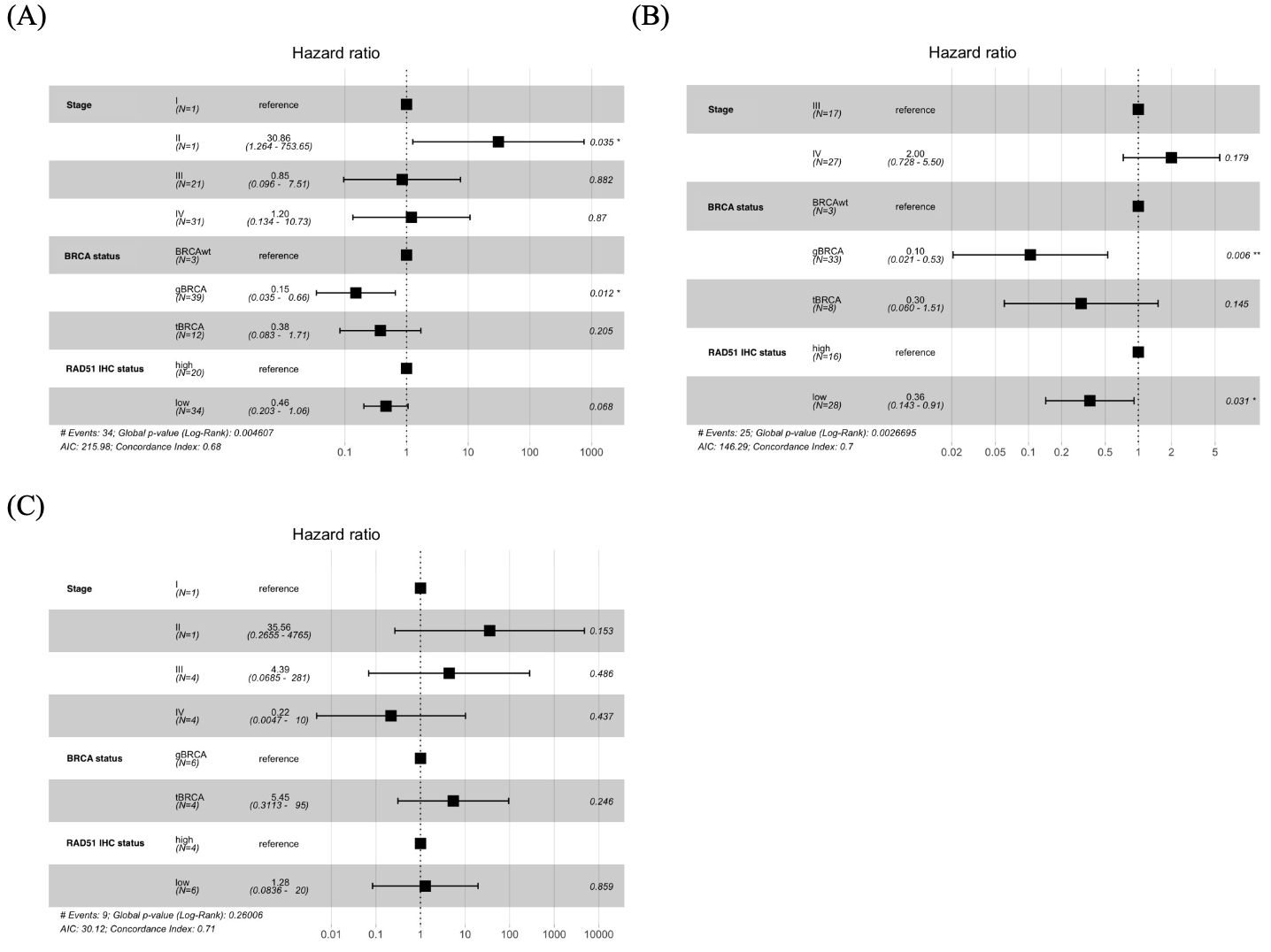
**

**Supplementary Fig. S4.** Association between RAD51 IHC status and the GSS. (A) GSS with respect to RAD51 IHC status in pre-PARPi and post-PARPi therapy samples. Correlation between RAD1 H-score and each component of the GSS, including (B) loss of heterozygosity, (C) telomeric allelic imbalance, and (D) large-scale state transitions.


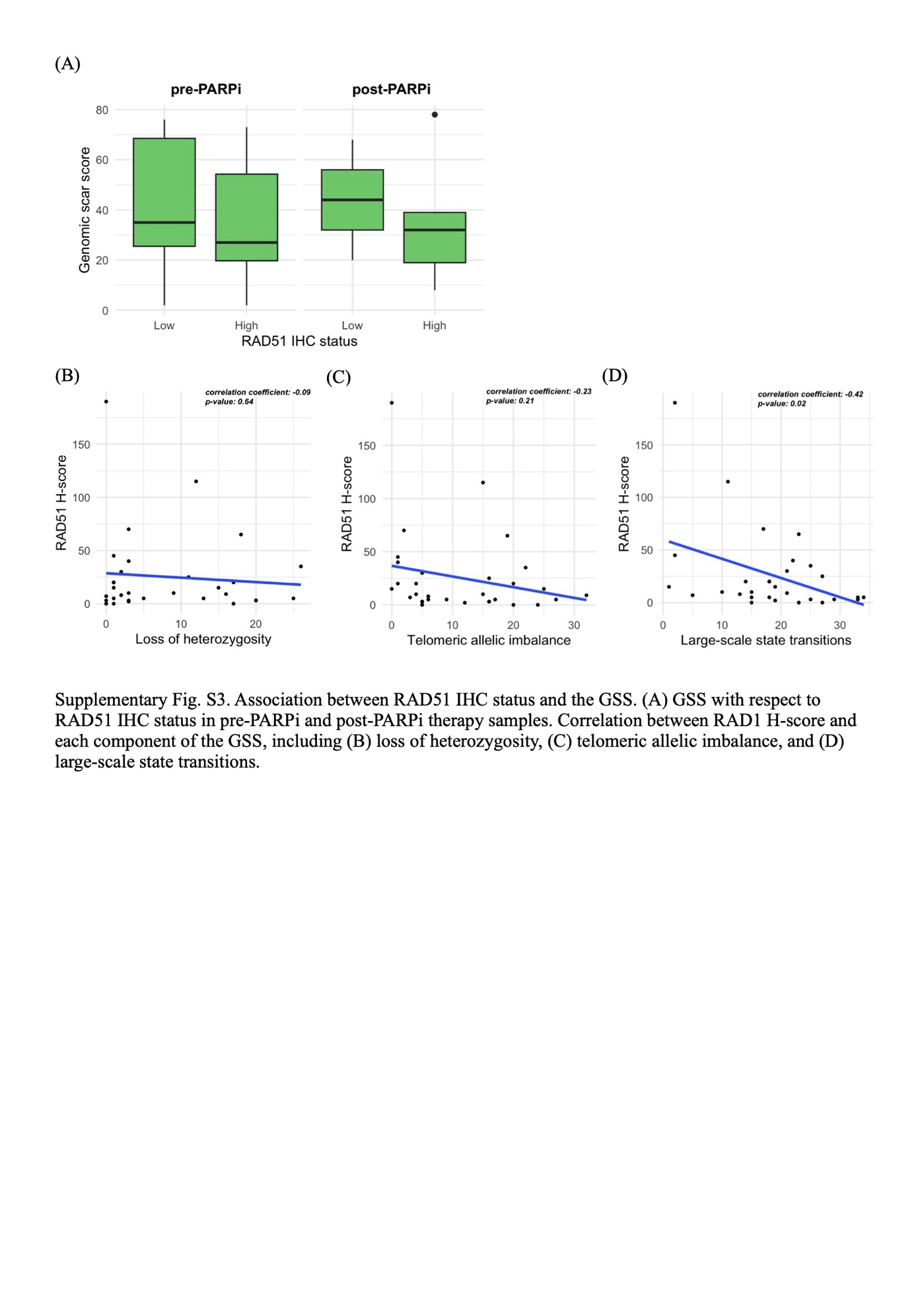


**Supplementary Fig. S5.** Mutation spectrum and classification of resistance mechanisms in post-PARPi ctDNA. Mutations, in homologous recombination (HR) restoration-associated genes, which were not present in pre-PARPi ctDNA, but newly acquired in post-PARPi ctDNA per patient, are marked with asterisks.


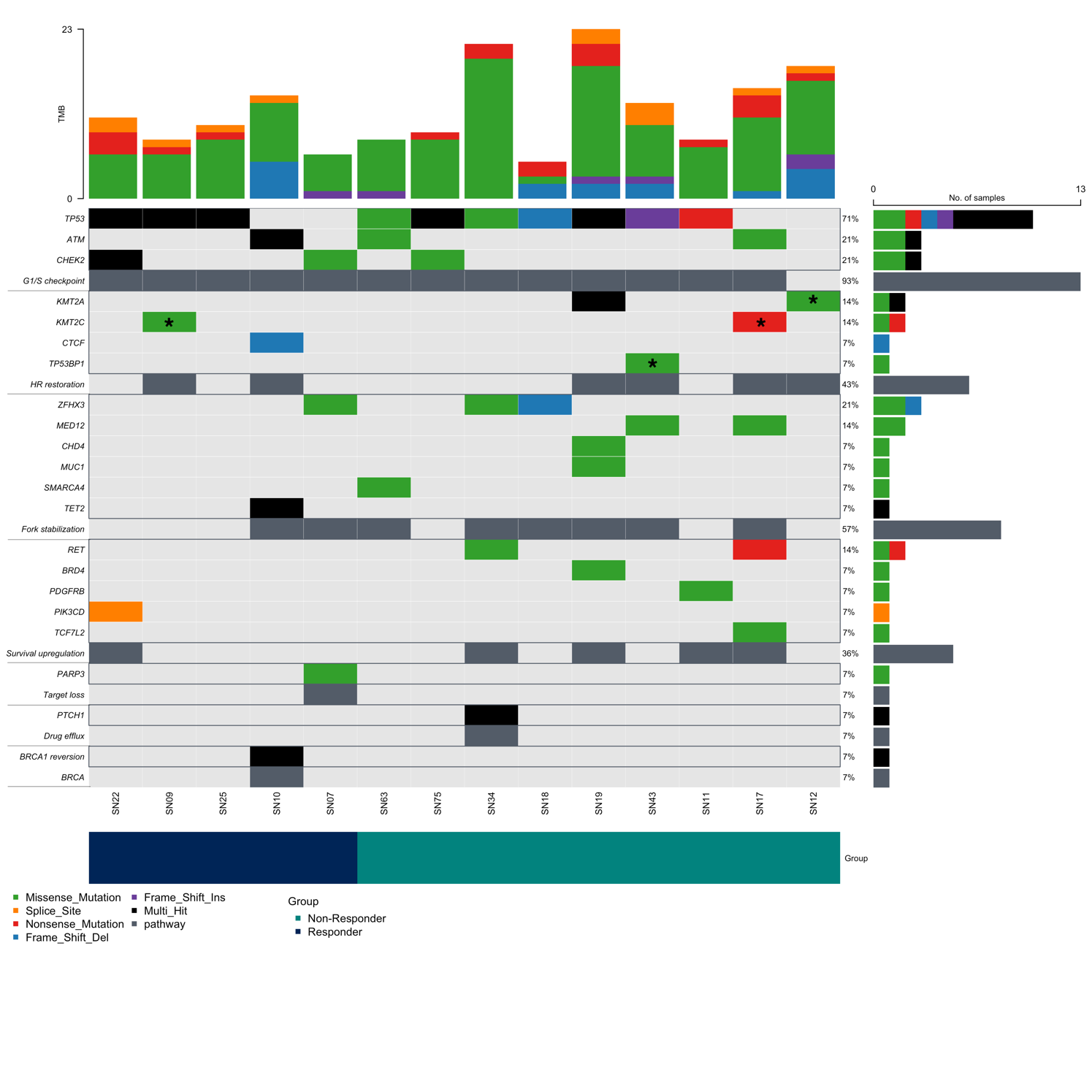

Supplement: Supplementary file 1 [file DataSheet_1.docx]
